# Celiac disease: quantity matters

**DOI:** 10.1007/s00281-012-0321-0

**Published:** 2012-06-26

**Authors:** Frits Koning

**Affiliations:** Department of Immunohematology and Blood Transfusion, Leiden University Medical Center, E3-Q, PO box 9600, 2300 RC Leiden, The Netherlands

## Abstract

Celiac disease (CD) is caused by uncontrolled immune responses to the gluten proteins in wheat and related cereals. Gluten is a complex mixture of gliadin and glutenin proteins and most modern wheat varieties contain up to 100 highly related, but distinct gluten proteins. Invariably, these gliadin and glutenin proteins contain immunogenic peptides, particularly so after the peptides have been modified by the enzyme tissue transglutaminase (TG2). This modification results in the conversion of glutamine residues in the gluten peptides into the negatively charged glutamic acid. This generates peptides that bind strongly to the disease predisposing HLA-DQ2.5 or -DQ8 molecules and this facilitates the induction of disease-inducing CD4 T cell responses, a hallmark of CD. It is well-known that the HLA-DQ genotype determines the risk of disease development. Moreover, the abundance of immunogenic peptides in the gluten proteins is likely linked to the observation that polyclonal T cell responses to multiple gluten peptides are usually found in patients with CD. However, not all patients respond to the same set of peptides. Here, I propose a model that integrates these observations and links them to the highly variable clinical spectrum of symptoms that are associated with CD. Moreover, I discuss whether it is feasible to alter wheat and/or gluten to make it suitable for consumption by CD patients.

## Introduction

Celiac disease (CD) is a chronic inflammatory disease of the small intestine [1, 2]. It can develop at any point in time during life in genetically susceptible individuals upon ingestion of wheat gluten and related cereal proteins. It is a frequent disorder affecting approximately 1 % of the population in the Western hemisphere [1, 2]. In the majority of patients, the disease goes in remission when patients are put on a gluten-free diet, and at present, a lifelong adherence to such a gluten-free diet is the only available treatment. CD shares important features with other autoimmune diseases: it is chronic, multifactorial, and with a female-to-male ratio of roughly 2 to 1 [1, 2]. Moreover, there is a strong HLA association, a compelling evidence that uncontrolled T cell responses are disease causing and the presence of autoantibodies is characteristic [1, 2]. Finally, a multitude of additional genetic loci have been identified that modulate the risk of disease development [3].

While it is unknown what initiates the development of CD, there is a unique insight into what drives the disease once it has been initiated. In the affected individual, four well-defined components form a “lethal” cocktail: gluten, tissue transglutaminase, HLA-DQ, and T cells. Upon ingestion of gluten-containing food products, the gluten is degraded into relatively large peptides by enzymes in the gastrointestinal tract [4]. Some of these peptides can bind directly to HLA-DQ2.5 (0501/0201) or -DQ8 (0301/0302) and trigger T cell responses which may result in local tissue damage and release of TG2 [5–7]. Subsequently, TG2 can modify a large number of gluten peptides [6, 8–14] which generates peptides that bind with much higher affinity to HLA-DQ2.5 or -DQ8, allowing an amplification of the gluten-specific T cell response. The release and subsequent activation of TG2 is thus a crucial step in disease development. Moreover, the gluten-specific T cell response leads to the release of inflammatory cytokines that induce local inflammation [15]. Concomitantly activation of intraepithelial lymphocytes takes place, driven by an increase in local IL-15 production [16–18], an event that is likely to contribute to the characteristic damage to the epithelial layer in CD patients. IL-15, a cytokine made by epithelial and dendritic cells, enhances the expression on intestinal epithelial cells of surface ligands (i.e., MICA and MICB) that are targets of the cytotoxic, NK-like IEL cells. Moreover, IL-15 upregulates the expression and function of NKG2D and thus plays an important role in the activation of the intraepithelial cell compartment [16–18]. Nevertheless, the very strong association between CD and HLA-DQ2.5/8 indicates that T cell responses to gluten peptides bound to HLA-DQ2.5/8 are a requirement for disease development. Significantly, such T cells are not found in healthy controls and a gluten-free diet is an effective treatment, lending further credit to the critical role of these gluten-specific T cells in the disease.

## Gluten peptides involved in disease pathogenesis

### Gluten is complex

**Fig. 1:**
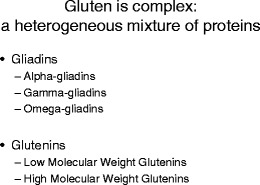
Overview of gluten gene families

Gluten is one of the most commonly used proteins in the food industry. Its characteristic properties make it an essential ingredient for the preparation of high quality dough, hence its popularity in the food (baking) industry [19]. Gluten is a complex mixture of gliadin and glutenin proteins (Fig. 1). The gliadin protein family contains α-, γ-, and ω-gliadins. The glutenin protein family contains low molecular weight (LMW) and high molecular weight (HMW) glutenins. While the LMW glutenins bear strong resemblance to the γ-gliadins, the HMW glutenins are distinct from the other gluten proteins. The HMW glutenins are largely responsible for the baking quality of dough [19].

### Wheat is complex

To make matters worse, wheat is also complex [19]. Over 10,000 wheat varieties are known worldwide. Through natural hybridization, tetraploid and hexaploid wheat varieties have evolved from ancient diploid varieties. While the tetraploid varieties are known as pasta wheat, the hexaploid varieties are used for the preparation of bread. In contrast to the ancient diploid species, that often yield only small amounts of gluten and are thus of no or limited commercial interest, the presently used commercial pasta and bread wheat varieties have been selected to produce large amounts of gluten and the expression of those HMW glutenins that are optimal for dough quality and baking properties [19]. Typically, commercial wheat varieties will express up to 100 distinct gluten proteins, the majority of which are gliadins and LMW glutenins.

### Immunogenic gluten peptides

Many studies have now documented the immunogenic nature of gluten (Table 1). In essence, three complementary approaches have been used to define the immunogenic sequences in gluten. In the first, reversed phase HPLC in combination with mass spectrometry was used to purify and identify immunogenic gluten peptides present in a crude gluten preparation [5, 10]. In the second, recombinant gluten proteins were produced to identify T cell stimulatory peptides present within those proteins [11]. In the third, large numbers of synthetic peptides were generated to encompass all known gluten sequences in the database and these were used to map the specificity of the gluten-specific T cell response [12, 20]. All three approaches have led to the identification of a multitude of gluten peptides that can stimulate T cells from patients. Strikingly, such peptides were found in α-, γ-, and ω-gliadins as well as in the LMW and HMW glutenins (Table 1). However, there is clear evidence for immunodominance. In HLA-DQ2.5-positive adults, responses to the α- and ω-gliadin-derived peptides are dominant, while responses to the γ-gliadins and LMW glutenins are (much) less frequently observed. In this respect, it is significant that the immunodominant α- and ω-gliadin peptides contain four proline residues while the γ-gliadin peptides have two or three and the LMW glutenin peptides only one or two (Table 1). It has been shown that the proline-rich nature protects gluten peptides from degradation in the gastrointestinal tract so that they will persist [4], increasing the chance that they will bind to HLA-DQ and trigger T cell responses. It is therefore tempting to speculate that this favors the survival of the particularly proline-rich α- and ω-gliadin peptides while the less proline-rich γ-gliadin and LMW glutenin peptides are degraded more rapidly, thus explaining the immunodominance of the α- and ω-gliadin peptides.

**Table 1:** List of celiac disease-relevant T cell epitopes; the nine amino acid cores are shown, nomenclature according to ref. 45

DQ2.5-restricted epitopes	
DQ2.5-glia-α1a	P F P Q P E L P Y
DQ2.5-glia-α1b	P Y P Q P E L P Y
DQ2.5-glia-α2	P Q P E L P Y P Q
DQ2.5-glia-α3	F R P E Q P Y P Q
DQ2.5-glia-γ1	P Q Q S F P E Q Q
DQ2.5-glia-γ2	I Q P E Q P A Q L
DQ2.5-glia-γ3	Q Q P E Q P Y P Q
DQ2.5-glia-γ4a	S Q P E Q E F P Q
DQ2.5-glia-γ4b	P Q P E Q E F P Q
DQ2.5-glia-γ4c	Q Q P E Q P F P Q
DQ2.5-glia-γ4d	P Q P E Q P F C Q
DQ2.5-glia-γ5	Q Q P F P E Q P Q
DQ2.5-glia-ω1	I P F P Q P E Q P F
DQ2.5-glia-ω2	P Q P E Q P F P W
DQ2.5-glut-L1	P F S E Q E Q P V
DQ2.5-glut-L2	F S Q Q Q E S P F
DQ2.5-hor-1	P F P Q P E Q P F
DQ2.5-hor-2	P Q P E Q P F P Q
DQ2.5-hor-3	P I P E Q P Q P Y
DQ2.5-sec-1	P F P Q P E Q P F
DQ2.5-sec-2	P Q P E Q P F P Q
DQ2.5-ave-1	P Y P E Q E E P F
DQ2.5-ave-1b	P Y P E Q E Q P F
DQ8-restricted epitopes	
DQ8-glia-α1	E G S F Q P S Q E
DQ8-glia-γ1a	E Q P Q Q P F P Q
DQ8-glia-γ1b	E Q P Q Q P Y P E
DQ8-glut-H1	Q G Y Y P T S P Q

Glutamic acid residues (E) formed by TG2-medited deamidation of Q residues in the native gluten sequences are shown

*glia-α* α-gliadin, *glia-γ* γ-gliadin, *glia-ω* ω-gliadin, *glut-L* low molecular weight glutenin, *glut-H* high molecular weight glutenin, *hor* hordein, *sec* secalin, *ave* avenin

The binding of gluten peptides to HLA-DQ2 and HLA-DQ8 has been studied in detail [14, 21–23], and the structure of alpha-gliadin peptides bound to HLA-DQ2 and HLA-DQ8 was determined [24, 25]. These results highlight the importance of negatively charged residues in gluten peptides for binding to HLA-DQ2/8. In particular, HLA-DQ8 prefers negatively charged residue at relative positions 1 and 9, while HLA-DQ2 favors such residues at positions 1, 4, and 6 in particular but to a lesser extent also at most other positions in the bound peptide [22]. While gluten contains almost no negatively charged amino acids, this is introduced due to the activity of the enzyme TG2 [8, 9]. TG2 selectively converts glutamine (Q) residues in gluten peptides into the negatively charged glutamic acid (E), largely based on the spacing between proline (P) and Q residues in gluten peptides: while in the sequence QXP (in which X is any amino acid), the Q is modified into E; this is not the case in the sequences QP and QXXP [13]. As these sequences are commonly found in gluten proteins, this allows an accurate prediction of which glutamine residues will be modified and which will not [13]. Strikingly, the most immunodominant gluten peptides all harbor a P at relative position 8 and a Q at position 6 which thus leads to the modification of the Q at position 6, introducing a strong anchor for binding to HLA-DQ2. Alternatively, the presence of a P and Q at positions 6 and 4, respectively, results in the introduction of an E at position 4, another strong anchor [22]. Similarly, an E is introduced at position 1 and/or 9 in HLA-DQ8-binding peptides. Thus, there is an unfortunate match between the specificity of TG2, the properties of gluten, and the binding requirements of HLA-DQ2 and HLA-DQ8 which results in the generation of highly immunogenic complexes between these HLA-DQ molecules and gluten peptides.

### HMW glutenins

An unresolved issue is the immunogenic nature of the HMW glutenins. To date, only one immunogenic peptide has been found in these proteins and this peptide is only recognized by T cells from HLA-DQ8-positive patients [7]. However, the peptide involved is a repetitive sequence in the HMW glutenins and 32 variants exist, most of which are immunogenic [7]. As a sizable proportion of HLA-DQ2-positive patients will also express HLA-DQ8, this peptide may thus cause problems in such patients as well. Yet, it is clear that the frequency of T cells reactive with this peptide is low [20]. With respect to HLA-DQ2, only one report has indicated that the HMW glutenins are also immunogenic, but the nature of the peptide(s) involved has not been elucidated [26]. Therefore, more work is needed to establish if the HMW glutenins pose a problem to patients, an issue that is relevant as the HMW glutenins in particular are essential for the generation of high quality dough [19].

## T cell cross-reactivity

CD patients are usually intolerant to wheat, barley, and rye while many tolerate oats. Barley, rye, and oats contain proteins that are homologous to gluten and these are called hordeins, secalins, and avenins, respectively. It is thus logical to assume that the intolerance to wheat, barley, and rye is based on T cell responses to gluten, hordeins, and secalins, respectively. Indeed, T cell responses to all these proteins can be observed in CD patients [20, 27]. Strikingly, while T cells can be highly specific for a peptide derived either from gluten, hordein, or secalin, others are broadly cross-reactive and respond to homologous peptides derived from all three cereals [20, 27]. Obviously, such cross-reactive T cells will respond upon ingestion of any of these three cereals and may thus be particularly deleterious in the patients with the disease.

The safety of oats for CD patients is still a matter of controversy. Clearly, the avenins are homologous to gluten but much less so then the hordeins and secalins. In fact, only two avenin peptides have been found to stimulate T cells from patients [27, 28]. Combined with the low abundance of avenin proteins in oats, this implies that the exposure to immunogenic peptides upon oats consumption is significantly lower compared to that after consumption of wheat, barley, and/or rye and apparently insufficient to induce disease symptoms in the large majority of patients. Nevertheless, occasionally patients cannot tolerate oats [29] and this must be taken into account when considering the introduction of oats in the gluten-free diet.

## HLA-DQ2 versus HLA-DQ8: quantity matters

**Fig. 2:**
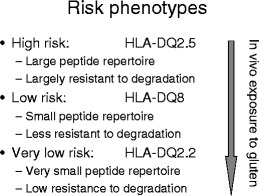
Correlation between the risk of HLA-DQ genotypes, the associated gluten peptide repertoire, and resistance to degradation by gastrointestinal enzymes

For HLA-DQ2.5, a large number of immunogenic peptides have been described (Table 1) and the strong association of CD with HLA-DQ2.5 may be related to the presence of this large repertoire of gluten peptides, many of which are resistant to degradation by gastrointestinal enzymes. In contrast, for HLA-DQ8, only a limited number of peptides have been found and, strikingly, the proline content of those peptides varies between 1 and 3, suggesting that they are more easily degraded than the immunodominant HLA-DQ2 epitopes. Thus, the weak association of CD with HLA-DQ8 might be explained by a smaller repertoire of immunogenic peptides that is less resistant to degradation. However, it should be noted that for HLA-DQ8, there is no correlation between proline content and immunodominance as the α-gliadin peptide EGSFQPSQE contains only one proline yet is most commonly recognized by T cells from HLA-DQ8-positive patients. Other factors may thus determine the immunodominant nature of this peptide. Nevertheless, the available data indicate that the level of gluten presentation may have a strong impact on the chance of disease development. There is in fact additional evidence that supports this concept. First, it is known that HLA-DQ2.2 (0201/0202) does not predispose to CD whereas the highly related HLA-DQ2.5 does. We have previously shown that while HLA-DQ2.5 can bind and present the full repertoire of gluten-derived peptides, HLA-DQ2.2 can only bind and present a few of these [21]. In agreement with this, a recent study has shown that in rare HLA-DQ2.2 + HLA-DQ2.5-CD patients, only T cells reactive to two gluten peptides could be found [30]. Significantly, one of these peptides was an already known LMW glutenin peptide [6] that contains only two prolines and is thus likely more rapidly degraded compared to other gluten peptides. Second, HLA-DQ2.5 homozygous individuals have an at least fivefold higher risk of disease development compared to HLA-DQ2.5/x heterozygous individuals, and we have shown that this is linked to a much more efficient presentation of gluten peptides due to the higher expression of HLA-DQ2.5 on antigen-presenting cells [21]. Finally, as pointed out above, oats is tolerated by the majority of patients despite the presence of two immunogenic peptides. Altogether, this indicates that quantity matters: the higher the exposure to immunogenic HLA-DQ–gluten peptide complexes, the higher the probability of disease development (Fig. 2).

## Children versus adults

A still unresolved issue is whether the repertoire of gluten-specific T cells in children is distinct from that in adults. While immunodominant responses to the α- and ω-gliadin peptides are found in adults [11, 12, 20], in children, responses to the LMW glutenins and γ-gliadins were frequently observed [6]. Moreover, in adults, mainly T cell responses to deamidated gluten peptides are found, but in children, responses to native gluten peptides were also relatively frequent [6]. Unfortunately, at present, no second study has been performed to confirm these results. However, previously, we speculated that these results may indicate that early in disease development a broad gluten-specific T cell response develops that can be directed to any of the immunogenic gluten peptides [6]. Furthermore, the observed response to non-deamidated peptides in children may indicate that this initial response is triggered by native gluten peptides. Importantly, the release of IFNγ will increase the expression of HLA-DQ on the cell surface of antigen-presenting cells allowing more efficient presentation of gluten peptides and thus amplifying the gluten-specific T cell response. Moreover, the (low level) inflammation would lead to the release and activation of TG2 that will broaden the repertoire of immunogenic gluten peptides by generating neo-epitopes that bind with high affinity to HLA-DQ, a second amplification loop. Eventually, the T cell response will focus on the most stable and immunogenic gluten peptides: deamidated peptides from α- and ω-gliadin that bind with high affinity to HLA-DQ. In such a scenario, the difference between children and adults reflects the maturation of the gluten-specific T cell response in time after loss of tolerance to gluten.

## Model

**Fig. 3:**
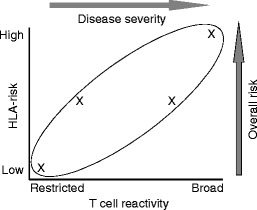
The combination of diversity in the T cell repertoire against gluten peptides and HLA-risk phenotype may underlie the diversity in symptoms associated with CD. For explanation, see the main text

CD has many faces. Symptoms vary dramatically between patients, and while some are highly sensitive to gluten, others are more or less tolerant, like the so-called silent patients. In fact, the typical clinical picture associated with childhood CD is quite rare and most patients present otherwise. Clearly, many factors may be responsible for this variety, including currently unknown genetic and environmental factors, but two known factors are worth considering: the HLA-DQ genotype and the nature of the gluten-specific T cell response. It is currently widely accepted that the risk of development of CD is largely determined by the HLA-DQ genotype (Fig. 2), but there is no clear correlation between the HLA type and the severity of the disease and associated symptoms. Nevertheless, individuals homozygous for HLA-DQ2 can present gluten peptides more efficiently and this could contribute to disease severity as well. In addition and as outlined above, T cell responses to a variety of gluten peptides are found in patients with CD. Although it is extremely difficult to quantify these responses, it is clear that not all patients respond to the same panel of gluten peptides, and while some of those responses are directed to relatively easily degraded γ-gliadin and LMW glutenin peptides, others are specific for the degradation resistant α- and ω-gliadin peptides. The ratio between T cells specific for α/ω- and γ/LMW peptides in the individual patient may thus (co-)determine the strength of the immune response upon gluten ingestion. Also, some T cells are highly specific for particular gluten peptides but others cross-react with homologous peptides in cereals and the ratio between these T cells may vary from patient to patient. It is therefore tempting to speculate that the actual repertoire of the gluten-specific T cells in the individual patient is linked to the severity of the symptoms in the large majority of patients: those that have an intermediate HLA-risk genotype (Fig. 3).

## The “holy grain”: is it possible to generate safe wheat?

The presence of many immunogenic sequences in wheat gluten severely complicates the generation of wheat that would be safe for consumption by CD patients. Commercial wheat varieties including bread wheat, pasta wheat, spelt, and triticale (a cross between wheat and rye) all contain a multitude of gluten proteins, each encoding one or more immunogenic sequences. Extensive studies have demonstrated that it is possible to identify wheat varieties that have a more favorable “toxicity” profile compared to other varieties, meaning that they contain less immunogenic peptides [31–36]. But none will be completely safe for patients. The generation of truly safe wheat would thus involve either the complete knock down of the expression of all gluten proteins or the modification of all gluten genes so that they would encode gluten proteins that are safe for consumption by patients (see also below). While it is indeed possible to knock down the expression of gluten genes [37], such an approach would yield wheat that has lost many of the properties that make it so suitable for the production of food products. The alternative approach requires the modification of dozens of gluten genes at least, a virtually undoable task. Therefore, neither of these approaches is an attractive option at present.

## Generation of safe gluten

It is known that much diversity exists between the individual members of gluten gene families. For example, over 3,000 alpha-gliadin genes are present in the gluten database and these genes encode a multitude of related yet distinct proteins. Large-scale analysis of these gliadin proteins has indicated that not all are equally “toxic” as the number of T cell stimulatory peptides present varies [38]. In fact, the gliadin proteins from the D-genome, present in bread wheat but not in pasta wheat, were found to be the most deleterious [38]. Not only do they contain all four known T cell stimulatory peptides, many of them also harbor a repeat of the glia-α1 and glia-α2 peptides that is commonly known as the highly immunogenic 33-mer. In contrast, gliadin proteins from the A- and B-genome contain only one or two of the known epitopes and lack the (full length) 33-mer repeat sequence, indicating that they would potentially be less harmful for CD patients [38]. Yet, none of the 3,000 genes analyzed encoded a protein that would be entirely safe, further indicating that it is impossible to generate truly safe wheat through conventional breeding strategies [38]. However, further analysis of the gliadin genes indicated that many variants exist of the known immunogenic α-gliadin epitopes encoded by the D-genome. Subsequent analysis of the HLA-DQ2-binding capacity and T cell stimulatory properties of these variant peptides indicated that not all these peptides bind equally well to HLA-DQ2 and that several lacked T cell stimulatory capacity despite proper binding to HLA-DQ2 [38]. Strikingly, many of the latter variants were found to harbor a serine (S) at relative position 3 or 8 in the peptide where the agonist peptide had a P. Importantly, all three immunogenic HLA-DQ2 epitopes encoded by the D-genome harbor a P at both positions 3 and 8 and substitution of these indeed eliminated their T cell stimulatory capacity. Similarly, variants of the 33-mer could be constructed that completely lacked T cell stimulatory capacity. Moreover, other natural variations were discovered that eliminated the immunogenic nature of α-gliadin peptides that are presented by HLA-DQ8. These results indicate that detoxification of α-gliadin proteins could be achieved by the introduction of naturally occurring variations in α-gliadin genes such that a selected P residue is replaced by an S. Importantly, as these are minimal and naturally occurring modifications, such proteins will have retained many of the properties that are unique to gluten proteins and essential for proper formation of high quality dough. It is likely that similar approaches can be used to produce other detoxified gluten proteins, like the HMW glutenins. Thus, the production of safe gluten appears to become a realistic option. However, for application in the food industry, such safe gluten proteins need to be produced in large quantities in a cost-effective manner. This requires the generation of transgenic crops, for example transgenic rice or corn in which the modified gluten genes are expressed. While such approaches are feasible, one has to take into consideration the regulatory issues involved and the public opinion concerning transgenic crops, especially in Europe. However, it is important to point out that while most transgenic crops currently in use have been generated for the benefit of the producer, crops that would produce safe gluten would benefit the consumer and the celiac society in particular. They may thus be more acceptable to both regulatory authorities and the general public.

## Enzymatic detoxification of gluten

Alternative methods to detoxify gluten proteins have also been explored. First, the degradation of gluten proteins during the food processing process appears to be a viable option. Extensive proteolytic degradation of gluten proteins can be obtained by sourdough fermentation and the products generated are well tolerated by patients [39].

Another approach is to use enzymes that efficiently cleave the glutamine- and proline-rich gluten proteins in the gastrointestinal tract. Such enzymes include a cysteine endoprotease isolated from barley [40], a prolyl endopeptidase from *Sphingomonas capsulata* [41], and a prolyl endoprotease from *Aspergillis niger* [42]. The former two are currently tested in a clinical trial (www.alvinepharma.com) and the latter has been proven to effectively degrade gluten under simulated gastrointestinal conditions [43]. Oral application of such enzymes may thus aid in gluten degradation in vivo, for example when eating out or traveling—situations when inadvertent gluten exposure can occur.

Alternatively, the use of enzymatic modification has been investigated [44]. In the small intestinal tract, gluten is modified by the enzyme TG2 which converts particular Q residues into the negatively charged E, creating peptides that bind with high affinity to either HLA-DQ2 or -DQ8. Alternatively, TG2 can crosslink the Q to a K (lysine) residue in another protein which would effectively block the ability of such gluten proteins to stimulate T cells. Indeed, Gianfrani and colleagues [44] were able to demonstrate that treatment of gluten with a microbial transglutaminase and lysine methyl ester yielded modified gluten that had virtually lost its capacity to stimulate gluten-specific T cells in vitro. As microbial transglutaminases are commonly used in the food industry, this may offer an attractive means to mass produce gluten proteins that are safe for consumption. In vivo safety, however, still has to be demonstrated in a clinical trial.

## Implications

Once CD has developed, exposure to even very small amounts of gluten can cause problems, hence the need for a strict gluten-free diet. However, I have argued that quantity matters: the higher the exposure to immunogenic HLA-DQ–gluten peptide complexes, the higher the chance that CD will develop. How can we use this knowledge? Perhaps we should reconsider the way in which gluten is introduced into the diet. Current guidelines indicate that gluten may be introduced into the diet 6 months after birth. This introduction is rather abrupt and 1-year-old children consume between 6 and 9 g of gluten daily. Considering that gluten contains a multitude of immunogenic sequences, one wonders if this is a smart thing to do. After all, you provide the immune system with ample opportunity to respond to the immunogenic gluten that passes the gastrointestinal tract on several occasions daily. Especially under conditions that promote the induction of inflammatory T cell responses, for example during infections in the gastrointestinal tract, this may lead to the breaking of tolerance to gluten. The release of IFNγ by pathogen-specific T cells may be the first step towards breaking oral tolerance to gluten as it would increase HLA expression on antigen-presenting cells and thus increase the level of gluten presentation. Simultaneous release of active TG2 due to local tissue damage would increase the repertoire of gluten peptides that can be presented and thus work in concert with the increased HLA expression. When naive gluten-reactive T cells would be present in the mesenteric lymph nodes under these conditions, one can envisage that the pro-inflammatory milieu would lead to their activation and ultimately to loss of tolerance to gluten. Little is presently known about the precursor frequency of gluten-reactive T cells in healthy individuals except that the failure to isolate such cells from peripheral blood and small intestinal biopsies from non-celiacs suggests that they are rare. Perhaps this indicates that CD is only initiated when three factors coincide: (1) a high level of gluten exposure, (2) a gastrointestinal infection, and (3) the presence of sufficient naive gluten-reactive T cells in the local mucosal tissue. This would explain why CD can develop at any age in life: only when these requirements are met disease develops and this will not necessarily happen at a particular time during life. The third factor, the generation of gluten-reactive T cells, cannot be controlled as the development of potentially gluten-reactive T cells is the result of positive and negative selection of T cell receptors in the thymus. However, we can influence to a certain extent for the first two factors, the level of gluten exposure and gastrointestinal infections. Reduction of gluten intake and/or more gradual introduction of gluten into the diet can in principle easily be achieved, especially in families at risk where the danger associated with gluten is recognized. Moreover, if the current vaccination programs to protect against childhood rotavirus show an impact on the prevalence of CD, this could indicate that gluten intake should be avoided when there are signs of gastrointestinal infections. It might also lead to initiatives to reduce gluten intake in the general population. The latter, however, will be hard to achieve as wheat is one of the largest crops in the world and wheat-based products are an almost inseparable component of the diet of the general population in the Western hemisphere. It will be hard to convince the food industry and non-celiacs that they will have to replace good-tasting food products for alternatives when they do not see any benefit. So at present, prevention of CD might be achieved by reduction of gluten intake and monitoring of gastrointestinal infections, approaches that can only be implemented in families at risk.

Finally, why do so few people have CD? We almost all eat large amounts of gluten, some 40 % of us are HLA-DQ2 and/or -DQ8 positive, and we all have gastrointestinal infections, yet only 1 % develops CD. What are we missing? Is there active protection, and if yes, through which mechanism? There is an obvious need to find out what licenses the induction of an effector T cell response to gluten as this leads to lifelong immunity to gluten. This is the challenge that lies ahead.

In conclusion, there is ample evidence that CD is caused by inflammatory T cell responses to gluten-derived peptides. Gluten contains a multitude of immunogenic peptides, and while some of those can stimulate T cell responses in their native form, most require modification by TG2. Such peptides specifically bind to either HLA-DQ2 or HLA-DQ8, explaining the strong association between CD and these HLA alleles. Quantity matters: the higher the level of gluten presentation, the higher the chance to develop CD. Reduction of gluten intake may thus be an effective approach to prevent or delay the development of CD. This is particularly relevant in families where CD occurs. The high complexity of gluten gene families in both ancient and commercial wheat varieties at present prohibits the development of wheat that is safe for consumption by CD patients. There are possibilities, however, to detoxify gluten, either through recombinant technologies, enzymatic degradation, or by enzymatic modification. This may prove to be valuable additions to the gluten-free diet.
